# Patients with acute intracerebral hemorrhage and severe symptoms are highly sensitive to prehospital delay. A subgroup analysis from the RESIST and TRIAGE-STROKE trials

**DOI:** 10.3389/fstro.2024.1437746

**Published:** 2024-08-21

**Authors:** Anne Behrndtz, Claus Z. Simonsen, Jan B. Valentin, Grethe Andersen, Rolf A. Blauenfeldt

**Affiliations:** ^1^Department of Neurology, Physiotherapy and Occupational Therapy, Gødstrup Hospital, Herning, Denmark; ^2^Department of Neurology, Aarhus University Hospital, Aarhus, Denmark; ^3^Danish Center for Health Services Research, Department of Clinical Medicine, Aalborg University and Aalborg University Hospital, Aalborg, Denmark; ^4^Department of Clinical Medicine, Aarhus University, Aarhus, Denmark

**Keywords:** stroke, prehospital, intracerebral hemorrhage, delay, triage

## Abstract

**Background:**

Patients with a positive prehospital stroke severity score and underlying intracerebral hemorrhage (ICH) may be harmed by longer onset-to-admission time. We aimed to investigate the interaction between ICH severity and time from onset to admission on functional outcome.

**Methods:**

This is an individual patient data analysis with data from two randomized prehospital stroke trials using the same prehospital stroke scale. Patients were stratified according to the presence of a positive stroke severity score. They were grouped into early arrivers (admitted ≤ 90 min from onset) and late arrivers (admitted ≥90 min after onset). The primary outcome was a shift toward a better functional outcome on the modified Rankin Scale (mRS).

**Results:**

A total of 212 patients had ICH. A positive stroke severity score was seen in 123 of these patients. Patients with ICH and a positive prehospital stroke severity score had a significantly worse outcome if they arrived 90 min or later at the hospital (adjusted odds ratio [aOR]: 2.02, 95% CI [1.01, 4.12]). This difference was not observed in patients without a positive severity score (aOR: 0.50, 95% CI [0.22, 1.14]). Patients with a positive score also had an increased risk of death or severe dependency (mRS of 5–6) of 9.1 percentage points (95% CI [−1.6%, 19.8%]) per hour if they were diagnosed with ICH.

**Conclusion:**

Longer onset-to-admission time was harmful for patients with ICH and a positive prehospital stroke severity score.

## Background

Guidelines recommend using a prehospital stroke severity score because patients eligible for thrombectomy often present with severe neurological deficits (McTaggart et al., 2020; Ramos et al., 2021). The functional outcomes after intravenous thrombolysis and endovascular therapy (EVT) in patients with acute ischemic stroke (AIS) are highly time-sensitive, whereas the outcomes for patients with intracerebral hemorrhage (ICH) have traditionally been considered time-insensitive (Saver et al., 2016). The Effect of direct transportation to thrombectomy-capable center vs. local stroke center in patients with suspected large vessel occlusion stroke in nonurban areas (RACECAT) trial included patients with a positive prehospital severity scale score (de la Ossa et al., 2022). Unexpectedly, bypassing a primary stroke center (PSC) for faster EVT for patients with AIS provided no benefit. In a subsequent analysis, the researchers found that patients with ICH were harmed by the longer transport time (Ramos-Pachón et al., 2023).

We aimed to investigate how prehospital delay affects outcomes for patients with ICH and whether how patients performed on a prehospital stroke severity scale modifies this.

## Methods

We conducted a *post*-*hoc* analysis of two recently completed prehospital stroke trials: the Remote ischemic conditioning for acute stroke trial (RESIST), trial (NCT03481777), which investigated the effects of remote ischemic conditioning in acute stroke, and the Transport strategy in patients with suspected acute large vessel occlusion stroke (TRIAGE-STROKE), trial (NCT03542188), which investigated the effects of bypassing a PSC (Behrndtz et al., 2023; Blauenfeldt et al., 2023). Both randomized controlled trials included patients that were able to arrive at a hospital < 4.5 h from symptom onset, and both trials scored patients using the same prehospital stroke severity scale registered on scene. In the RESIST trial, patients with both severe strokes and less severe strokes were included.

The two trials were approved as acute research studies, and consent was waived in the acute phase.

For this analysis, we included patients who arrived at the hospital within 300 min from symptom onset. The data, including demographic data, comorbidities, time measures, and data regarding the stroke, were prospectively collected. The prehospital stroke severity score used in both trials was the Prehospital Assessments Stroke Severity (PASS) score. PASS examines the presence of arm paresis, gaze palsy, and incorrect “level of consciousness” questions. A positive stroke severity score was defined as a PASS score of 2 or 3. The exposure was time from onset to admission, and for the primary analysis, the exposure was dichotomized and defined as 0–90 min vs. 90–300 min. The rationale behind this time limit was based on the findings from the RACECAT study, where the median time for the group admitted to the PSC was 94 min.

The primary outcome was a better neurological outcome measured as a shift toward a better functional outcome on the modified Rankin Scale (mRS) score 90 days after stroke. Secondary outcomes were independence (mRS score 0–2) and severe dependence or death (mRS score 5–6), National Institutes of Health Stroke Scale (NIHSS) and Glasgow Coma Scale (GCS) scores at arrival, the number of bed days, and the use of neurosurgery.

### Statistical analysis

We conducted descriptive statistics comparing late-arrival to early-arrival ICH patients and stratified by stroke severity. The primary endpoint was analyzed by applying ordinal logistic regression to the range of the mRS scores to estimate odds ratios (OR) for a shift toward better outcomes of early-arrival vs. late-arrival ICH patients. Second, a binary regression was applied to estimate the relative risk (RR) of the dichotomous outcomes for late-arrival vs. early-arrival ICH patients. Both analyses were stratified by stroke severity. The analysis was conducted after unadjusting and adjusting for age, comorbidity (atrial fibrillation, diabetes, hypertension), pre-stroke mRS score (living independently or not), and prehospital stroke severity score (2 vs. 3). Finally, we estimated the added risk of the dichotomized endpoints for the hourly increase in time from onset to arrival using linear regression with a robust variance estimation. The analysis was stratified by stroke severity and diagnosis (ICH and AIS) and repeated for the added chance of dependency and death (mRS 5–6) and a good functional outcome (mRS 0–2). The results were presented with 95% confidence intervals (CIs) where appropriate. All analyses were conducted in R (R Core Team [2022]. R: A language and environment for statistical computing. R Foundation for Statistical Computing, Vienna, Austria (URL https://www.R-project.org/).

## Results

**Figure 1:**
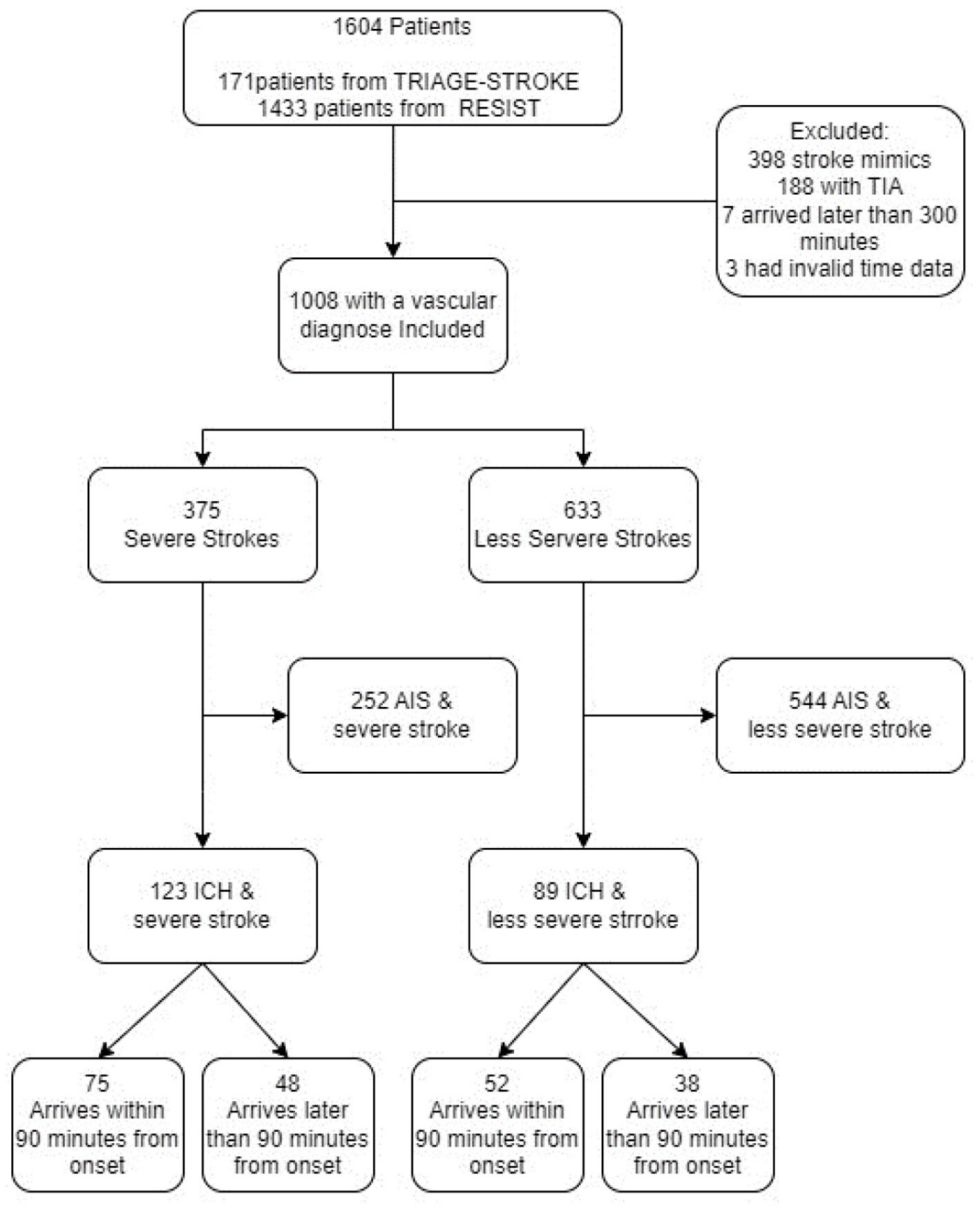
Consort diagram of the included patients in the trial. TRIAGE-STROKE, Transport strategy in patients with suspected acute large vessel occlusion stroke; RESIST, Remote ischemic conditioning for acute stroke trial; TIA, transient ischemic stroke; AIS, acute ischemic stroke; ICH, intracerebral hemorrhage.

We included 1,604 patients (1,433 from the RESIST trial and 171 from the TRIAGE-STROKE trial). We excluded all those with stroke mimics (*n* = 398), transient ischemic attacks (*n* = 188), arrival times longer than 300 min (*n* = 7), and invalid time data (*n* = 3) from further analyses. Of the 1,008 patients who had a confirmed stroke diagnosis, 796 were patients with AIS and 212 were patients with ICH. The median age was 72, and 44% were women. A study flow chart is presented in Figure 1. No significant differences in baseline characteristics were found between early and late arrivals for either group of patients regardless of their prehospital severity score.

**Figure 2:**
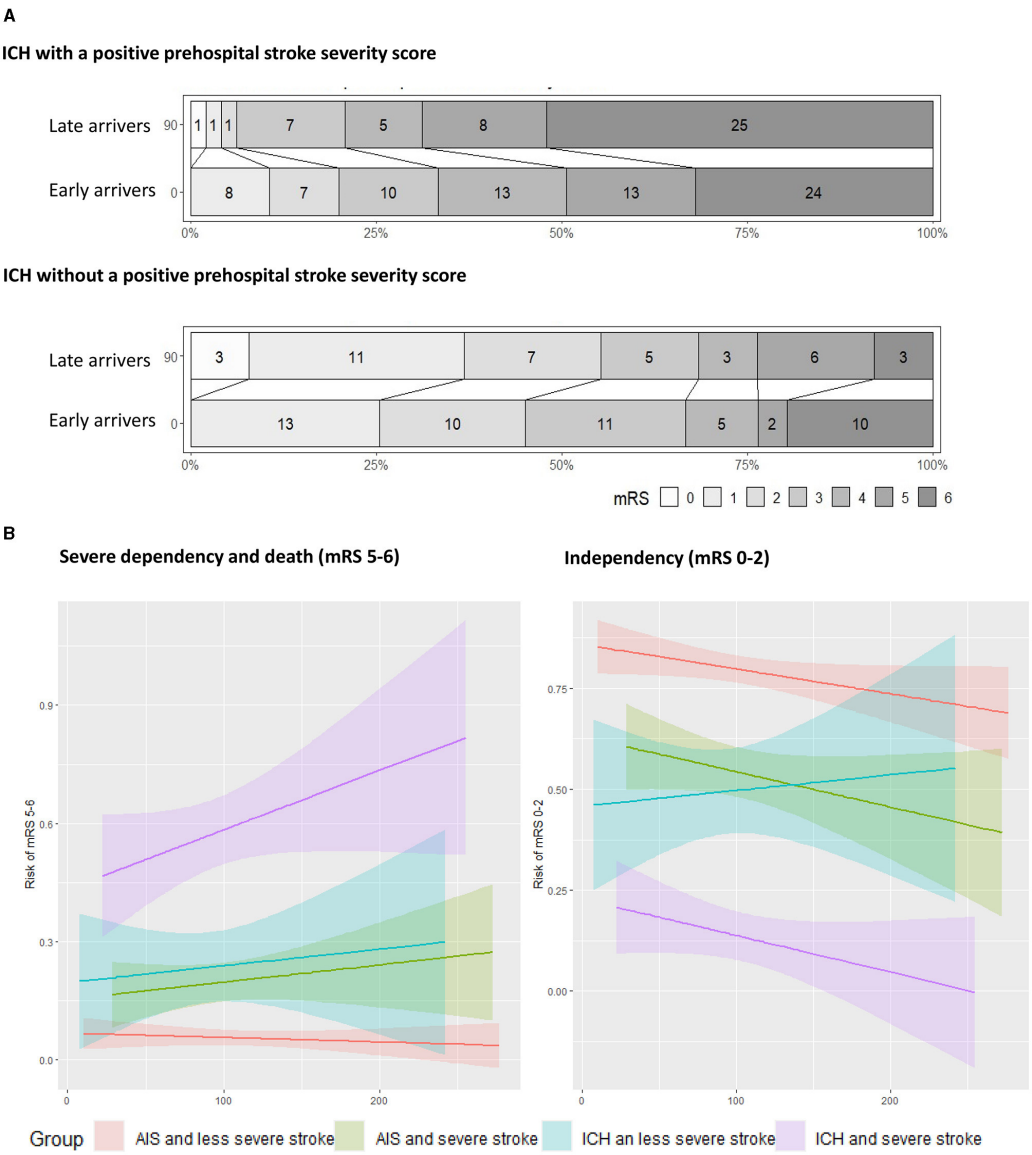
**(A)** Shows the modified Rankin Scale (mRS) outcomes as Grotta bars showing a shift toward better outcomes of patients with intracranial hemorrhage (ICH). Patients with a positive stroke severity score in the field arriving earlier than 90 min have significantly better outcomes at 90 days' follow-up, while patients without a positive score are not significantly harmed by the onset admission time. **(B)** Patients with ICH and a positive prehospital severity score have 9 percentage point increased risk of severe dependency and death for each hour of increased transport time. This increase is only 2.5% per hour for those with acute ischemic stroke (AIS) and a positive severity score. For those with ICH and a low severity stroke, admission time does not seem to have a harmful effect on patient independency at follow-up.

In the group of patients who had ICH and a positive prehospital stroke severity score, the OR for a shift to a lower (better) score on the mRS when comparing early to late arrivers was 2.28 (95% CI [1.17, 4.52]), and the adjusted OR was 2.02 (95% CI [1.01, 4.12]). In patients without a positive prehospital stroke severity score, no statistically significant differences were found when comparing early to late arrivers, with an OR of 0.63 (95% CI [0.29, 1.33]) and an adjusted OR of 0.50 (95% CI [0.22, 1.14]; Figure 2A and Table 1). Secondary dichotomized outcomes are shown in Table 1.

**Table 1:** Shows primary and secondary endpoints.

	**Positive prehospital stroke severity score** ***N*** = **123**			**Negative prehospital stroke severity score**, ***N*** = **89**		
	**Onset to arrival**<**90 min**, ***N*** = **75**	**Onset to arrival** >**90 min**, ***N*** = **48**	***p*** **-Value**	**Onset to arrival**<**90 min**, ***N*** = **51**	**Onset to arrival** >**90 min**, ***N*** = **38**	***p*** **-Value**
**Primary endpoint**						
Shift in mRS, OR (95% CI)	2.28 [1.17, 4.52]		0.015^*^	0.63 [0.29, 1.33]		0.23
Shift in mRS, adjusted OR (95% CI)	2.02 [1.01, 4.12]		0.048^*^	0.50 [0.22, 1.14]		0.10
**Secondary endpoints**						
Independency (mRS 0–2), RR [95% CI]	3.75 [1.15, 16.9]		0.046^*^	0.66 [0.28, 1.54]		0.3
Independency (mRS 0–2), adjusted RR [95% CI]	3.47 [0.97, 16.7]		0.077	0.52 [0.18, 1.41]		0.2
Severe dependency or death (mRS 5–6), RR [95% CI]	2.26 [1.07, 4.92]		0.036^*^	1.01 [0.37, 2.70]		>0.9
Severe dependency or death (mRS 5–6) adjusted, RR [95% CI]	1.97 [0.86, 4.66]		0.11	0.69 [0.20, 2.24]		0.5
**NIHSS, median (IQR)** ^‡^	17 (14, 21)	19 (15, 24)	0.2	8.0 (5.5, 13.5)	8.5 (5.0, 10.2)	0.4
**GCS, median (IQR)** ^§^	14.0 (11.0, 15.0)	12.0 (9.0, 14.0)	0.042^*^	15.00 (15.00, 15.00)	15.00 (14.00, 15.00)	0.12
**Number of bed days, median (IQR)** ^¶^	5 (2, 12)	5 (2, 8)	0.8	5 (3, 12)	7 (3, 9)	0.9
**Surgery**, ***n*** **(%)**	12 (16%)	5 (10%)	0.4	4 (7.8%)	3 (7.9%)	>0.9
**Delay**						
**Transport time, median (IQR)** ^†^	27 (19, 37)	48 (30, 69)	< 0.001^*^	25 (13, 33)	36 (28, 46)	< 0.001^*^
**Median onset to admission, median (IQR)**	64 (51, 78)	117 (104, 143)	< 0.001^*^	63 (48, 73)	125 (105, 153)	< 0.001^*^

OR, odds ratio; CI, confidence interval; RR, relative risk; IQR, interquartile range. ^*^Shift is analyzed with ordinal logistic regression for a better outcome if admitted earlier than 90 min. ^†^Randomization to admission is used as a proxy for transport time. ^‡^National Institute of Health Stroke Scale (NIHSS) score measured at admission. ^§^Glascow Coma Scale (GCS) score measured at admission. ^¶^Number of bed days at hospitals.

The GCS score at admission was significantly lower in the group of patients with ICH and severe stroke symptoms who arrived after 90 min compared to patients who arrived earlier (12 vs. 14, *p* = 0.042). No significant differences were observed in the number of bed days, NIHSS scores, or neurosurgical interventions in this group of patients, and no differences were observed in patients without a positive prehospital stroke scale score. However, in the group of patients with a positive prehospital score, the risk of getting a surgical intervention (EVD, craniotomy, or ICH evacuation) was 1.94 higher (95% CI [1.49, 2.55]) compared to those without a positive score (Table 1).

Figure 2B shows the dichotomized endpoints as a function of time for patients with and without a positive prehospital stroke severity score. The time dependence for patients with AIS is also shown. Patients with ICH and a positive prehospital stroke severity score had an increased risk of death or severe dependency (indicated by an mRS score of 5–6) of 9.1 percentage points (95% CI [−1.6%, 19.8%]) per hour for the first 300 min. Patients with ischemia and suspected severe stroke symptoms had an increased risk of death or severe dependency (indicated by an mRS score of 5–6) of 2.7 percentage points (95% CI [−3.1%, 8.4%] per hour. The odds of independence (mRS score 0–2) decreased by 5.4% (95% CI [−12%, 1.6%]) per hour for ICH patients with severe symptoms. As observed in the confidence intervals, none of these relations has proven statistically significant (Figure 2B).

## Discussion

This study combining two prehospital stroke trials showed that patients with a positive prehospital stroke severity score and a final diagnosis of ICH had significantly worse outcomes if they were admitted later than 90 min after onset. No significant difference existed for patients without a positive prehospital stroke severity score when comparing early to late arrivers, suggesting that these patients are not affected to the same extent as those with severe stroke symptoms and ICH. These results align with the findings from the prespecified subgroup analysis of the RACECAT trial, suggesting that the outcome after ICH may be highly sensitive to time to admission after onset (Outcomes and Registration, 2023).

This study highlights that patients with severe neurological deficits detected at scene or in ambulance remain a key target of clinical and scientific interest. Patients with ischemia have been shown to benefit from a faster time to admission and treatment with both thrombectomy and thrombolysis (Lees et al., 2010; Kaesmacher et al., 2020). We suggest that patients with severe deficits and ICH also benefit from faster admission at a hospital. The driver behind this finding remains uncertain. Aspiration, high blood pressure, and a continuation of anticoagulants and their effects have previously been suggested to be contributors to aggravating factors regarding outcomes (Ramos-Pachón et al., 2023).

No single treatment for ICH has shown convincing results regarding outcomes for these patients, but implementing a bundle of care for this group of patients has been proven effective on clinical worsening (Turner et al., 2015). The bundle consists of hemostatic treatment or anticoagulant reversal and treating aspiration, fever, and blood pressure. In the analysis of the patients with ICH from the RACECAT trial, more patients transported to comprehensive stroke centers had higher incidences of vomiting and pneumonia after their stroke (Ramos-Pachón et al., 2023). This could be a focus for further prehospital research and treatment because it may contribute significantly to the pathogenesis of clinical worsening of patients with ICH.

Acute hematoma expansion (HE) is a well-known reason for the clinical worsening of patients with ICH and frequently occurs within the 1^st^ h after onset; however, regarding HE, no significant difference was observed in the two arms in the RACECAT trial (Al-Shahi Salman et al., 2018; Ramos-Pachón et al., 2023). Hematoma volume and computed tomography markers are predictors of HE, and these markers may help select patients at the highest risk of HE who could benefit the most from a protective treatment (Brott et al., 1997; Demchuk et al., 2012; Boulouis et al., 2017). Two prehospital trials using mobile stroke units (MSUs) to investigate early treatment with hemostatic agents are ongoing (Naidech et al., 2022; Yassi et al., 2022). Worldwide, a limited number of patients are served by MSUs, emphasizing the need for diagnostic instruments that can be used by EMS, such as biomarkers or clinically validated instruments for ICH detection. We suggest that a positive prehospital stroke severity score is a predictor for a worse trajectory for patients with ICH.

To date, no randomized controlled trials have provided sufficient evidence that patients with AIS benefit from bypassing a PSC to get earlier EVT at a comprehensive stroke center (de la Ossa et al., 2022; Behrndtz et al., 2023). In this study, we saw that patients with ICH and severe symptoms were more sensitive to transport times than patients with AIS. Their rates of severe dependency and death increased by 9% per hour while the same rates for patients with AIS only increased by 2.5%. In the case of considering a bypass strategy, future triage models should take this into account, but one might speculate that patients with a positive score and ICH should be the target population for future interventions in trials.

This trial has several limitations. The RESIST trial investigated the effect of prehospital and in-hospital treatment with remote ischemic conditioning in acute stroke patients and was not designed to study optimal transport strategy; thus, residual confounding cannot be ruled out. In a few patients (*n* = 3), the hospital admission time was missing, but no data were missing for the primary endpoint. Nausea and vomiting were not prospectively collected for all patients from the prehospital files, and hematoma volume at baseline has not been estimated, which may limit the conclusions regarding the mechanism behind the observed differences. We acknowledge that, when displaying the linear associations between mRS scores and time (Figure 2B), these associations cannot be proven to be statistically significant.

We encourage that further prehospital research should focus on diagnosing and treating ICH with the same engagement as a previously conducted research differentiating large vessel occlusion among ischemic stroke patients.

## Conclusion

A longer transport time to the hospital has been indicated to harm patients with ICH and severe symptoms. Patients with ICH without a positive prehospital stroke severity score are not affected by onset to admission time.

## Data availability statement

Publicly available datasets were analyzed in this study. This data can be found here: accessible via corresponding author.

## Ethics statement

The studies involving humans were approved by Danish Research Ethics Committee, Central region Denmark and Northern region Denmark, (Hospitals of Hostebro, Aarhus and Aalborg). The studies were conducted in accordance with the local legislation and institutional requirements. The participants provided their written informed consent to participate in this study.

## Author contributions

AB: Conceptualization, Formal analysis, Investigation, Methodology, Project administration, Resources, Validation, Visualization, Writing – original draft, Writing – review & editing. CS: Funding acquisition, Investigation, Supervision, Writing – review & editing. JV: Data curation, Supervision, Validation, Visualization, Writing – review & editing. GA: Funding acquisition, Supervision, Writing – review & editing. RB: Conceptualization, Data curation, Investigation, Methodology, Supervision, Writing – review & editing.
